# Examination of Horses for Soundness1Read at meeting of the Virginia State Veterinary Medical Association, Staunton, Va., January 5, 1897.

**Published:** 1897-03

**Authors:** A. J. Burkholder


### THE EXAMINATION OF HORSES: ITS DIFFICULTIES AND RESPONSIBILITIES.[1]

By A. J. Burkholder, D.V.S.

The examination of horses as to soundness is a function in which every practitioner shares to a greater or less extent. Like many other callings it is one demanding for its efficient performance a large and varied experience of horses in general as well as a full share of that higher technical training which scholastic study affords. The responsibility which attaches to this department of our work is rapidly becoming more and more recognized and insisted upon by those in whose interest we act. The marked and

[1] Read at meeting of the Virginia State Veterinary Medical Association, Staunton, Va., January 5, 1897.

rapid increase in the quality of our horse-stock arising out of improved methods of breeding and a more painstaking system of rearing has very materially led to an increase in their value. With an increase in value there has very naturally arisen a desire on the part of the purchaser to guard against imposition and the many possible disappointments and losses which attach to the purchase of horses. It is mainly on this account that the services of the qualified veterinarian are now so much more in request than they were formerly; but there is also a decided tendency on the part of dealers to evade the responsibilities of "warranty" and to throw the entire responsibility upon the surgeon. This should be taken as a high compliment to the veterinary profession that the purchasers so readily accept this new state of things. By so doing they have declared confidence in the integrity and skill of our craft, and it is a matter of congratulation that whereas our law courts were formerly besieged by litigants seeking redress on account of real or supposed misrepresentations, such cases are now, comparatively speaking, rare.

I cannot help but feel that this more satisfactory state of relations between the vender and the purchaser is, in a large measure, due to the services rendered by our profession in the exercise of their judgment as to soundness, and upon which purchasers are now content to rely without any guarantee from the vender. It cannot, however, be overlooked, as previously observed, that this increase of public confidence has brought with it a corresponding increase in our responsibilities. Circumstances which have recently occurred in connection with several members of our profession have clearly demonstrated this. Serious claims have been set up against veterinarians on account of losses sustained by reason, as has been alleged, of their negligence or want of skill and judgment. And since the verdict of a jury is as uncertain as continuous health and soundness, we must feel a deep sense of insecurity in regard to this branch of our work, and it is to this fact that I wish to direct your attention.

No one who has had any experience in the examination of horses can have failed to recognize the trying circumstances under which we are often expected to exercise this branch of our calling. In no other profession is there a duty so peculiar in its nature or so precarious in its consequences. It is not with an inanimate machine that we have to deal—whose every part may be disconnected, scrutinized, and tested; but a living, complex, ever-changing organism, exposed to and influenced by ever-changing conditions of environment and purpose, and in a large measure also by the abuses of human vice and folly.

In proceeding to deal with this subject it is desirable at the outset to consider the nature of the duty involved, and in doing so we must first, I think, repeat the question so often asked, What is soundness? It has been laid down and is generally believed that "sound" means "perfect." To this definition I take no exception in the abstract; but if it is the standard to be recognized in dealing with horses, then it will be obvious at once that we can no longer hope to speak with authority on the subject.

The vital machinery of the animal organism is hidden away from us, and, except so far as objective physiological signs, cannot be scrutinized. I say, therefore, that this definition of soundness imposes upon us a duty and responsibility which we are not warranted in accepting. We cannot reasonably certify to the physical perfection or durability of any essential part that lies hidden from view because of the fact that slight structural changes or traumatic injuries may exist at the time of examination, which may soon afterward give rise to serious functional, systemic, or organic disease. Further, such a definition is so exclusive in its application to horses as practically to dispose of the necessity for any examination at all, as such a thing as perfection, even in the accessible parts of a horse at the working period of life, is quite phenomenal.

Many members of our profession seem to ignore this fact by claiming that soundness implies that an animal must be practically sound—that even if some abnormal condition exists which does not impair his usefulness, he must be considered sound. According to this definition, the meaning of soundness is the capability of an animal to perform such work as it is best fitted to perform by virtue of its size, build, and general character. This creates a vast discrepancy in the literal and legal sense of the term, and the unfitness of either to the requirements of a rational and consistent system of horse inspection and certification, which needs at this time our serious consideration. Shall we discard this portion of our practice on account of the litigation arising from such diversity of opinion, or shall we adopt a form of certification which by its literal and legal construction will not only relieve us from censure and responsibility, but afford our clients sufficient protection? The usual fee demanded for examination is wholly inadequate for any special warranty of soundness. Why, then, should we assume such a position? If we are forever afterward subject to costs incurred defending ourselves against persons who, by reason of their inability to place the proper value upon an animal, set up claims of unsoundness at the time of purchase, had we not better look well to our interest and obviate any trouble that might arise by misinterpretations and misconstructions by courts and juries? I will submit a form of certification which will, in my opinion, give to our clients all that should be expected from the hands of an honest, conscientious practitioner.

CERTIFICATE OF EXAMINATION.

Issued to Mr. upon the following-described animal:
Sex—gelding.
Age—six years.
Color—dark bay.
Points—two-inch star.
Scars—two-inch cicatrix on right forearm.
Height—15–1.
Breed—Hambletonian.
Class—American roadster.

Having made a careful and complete examination of the above-described animal, I hereby certify that I fail to detect any unsoundness.

Given under my hand, this 1st day of January, 1897.

That this form of certification admits of and encourages the practice of fraud and deception by unprincipled persons, I will not deny, but in all such cases redress can be had in our courts. The laws of our State are sufficiently severe on such culprits, and should be promptly enforced in all such cases; but where honesty of purpose prevails no responsibility should be implied. We cannot vouch for the honesty and integrity of the surgeon, neither can we assume to testify as to his skill in this important branch of our practice; but this risk should be taken by the client.

We are supposed to possess that higher degree of trained power of observation requisite to perfect knowledge of the anatomical structure and normal function of every part of the animal economy. This superior ability may be conceded to the most learned of our profession, but even the most skilled are often unable to meet the contingencies liable to occur under existing influences.

---

THE students' society of McKillip Veterinary College devotes part of its meetings to debate on topics of special interest in their work. Great interest is manifested in this College association, and its value to students is very noticeable in the increased interest shown in their studies.

